# Identification and characterization of SCC*mec* typing with *psm-mec* positivity in *staphylococci* from patients with *coagulase-negative staphylococci* peritoneal dialysis-related peritonitis

**DOI:** 10.1186/s12866-023-03017-2

**Published:** 2023-09-23

**Authors:** Jun Zhou, Chuishun Yang, Wenjuan Lei, Man Xu, Xingli Cai, Wanqiong Yuan, Hua Lin

**Affiliations:** 1 Department of Nephrology and Rheumatology, Haikou People’s Hospital Affiliated to Xiangya School of Medicine, Haikou, China; 2 Department of Orthopedics, Peking University Third Hospital, No. 49 North Garden Road, Haidian District, Beijing, China; 3 Beijing Key Laboratory of Spinal Disease, Beijing, China; 4 Engineering Research Center of Bone and Joint Precision Medicine, Beijing, China; 5 Department of Nursing, Haikou People’s Hospital Affiliated to Xiangya School of Medicine, 43 Renmin Road, Haidian Island, Haikou, China

**Keywords:** SCC*mec*, *Psm-mec*, Coagulase-negative *Staphylococci*, Peritoneal dialysis-related peritonitis, Peritoneal dialysis

## Abstract

**Background:**

Peritonitis is the most important complication of peritoneal dialysis (PD) and *coagulase-negative staphylococci* (CNS) are a frequent cause of dialysis-related infections. The association between SCC*mec* typing with *psm-mec* positivity in *staphylococci* and PD-related infections has not been identified. We aim to investigate the molecular epidemiology of CNS isolated from PD-peritonitis in a single Chinese center, focusing on the genetic determinants conferring methicillin resistance.

**Methods:**

We collected 10 genetically unrelated CNS isolates from 10 patients with CNS PD-related peritonitis. The patients were divided into two groups based on the results of MIC to oxacillin: the methicillin-resistant CNS (MRCNS) and methicillin-sensitive CNS (MSCNS) groups. The biofilm formation group (BFG) and the non-biofilm formation group (NBFG) were used as the control groups. Phenotypic and molecular methods were used to analyze SCC*mec* types I, II and III, associated genes and biofilm formation and the existence of *psm-mec*. The demographic data and clinical indicators were collected.

**Results:**

Ten CNS PD-related peritonitis patients were enrolled for this study. There were 6 MRCNS and 4 MRCNS isolates. SCC*mec* types were fully determined in 10 isolates. Seven *staphylococci* (70%) carried SCC*mec*, of which 4 isolates carried single SCC*mec* type I (40%) and 3 isolates had multiple SCC*mec* elements (I + III). Of the 6 MRCNS isolates, 3 carried SCC*mec* type I (50%) and 2 isolates carried SCC*mec* type I + III (33.3%). A high diversity of *ccr* types, *mec* complexes and *ccr-mec* complex combinations was identified among the 10 CNS isolates. The *psm-mec* gene was detected in 2/10 (20%) CNS isolates. There was no mutation in the *psm-mec* gene.

**Conclusions:**

The majority of isolates were hospital-associated isolates. Furthermore, 2 *psm-mec* positive isolates were MRCNS in the NBFG. The PD patients frequent exposure to hospital would be the main risk factor. The presence of the *psm-mec* signal in the spectra of the MRCNS tested here demonstrates the presence of certain SCC*mec* cassettes that convey methicillin resistance.

**Supplementary Information:**

The online version contains supplementary material available at 10.1186/s12866-023-03017-2.

## Introduction

Peritonitis is one of the most important complications of peritoneal dialysis (PD). It is still the main reason for patients to transfer from PD to hemodialysis [1]. Over the last few decades, the incidence of peritonitis caused by gram-positive pathogens in PD has decreased significantly, predominantly due to improvements in PD connectology system [2]. However, gram-positive peritonitis, particularly *Staphylococcus epidermidis* and other *coagulase-negative staphylococci* (CNS), remains a common cause of dialysis-related infections [3]. *Staphylococci* (*S*) *warneri*, *S. hominis*, *S.haemolyticus*, *S.saprophyticus*, *S.capitis* and *S.simulans* are *staphylococcal* species that have also been reported as infectious agents associated with PD [4]. Contamination at the time of PD fluid exchange is thought to be a major cause of peritonitis, mainly from external sources [5]. Many patients with CNS peritonitis have mild clinical symptoms. They respond well to outpatients treatment [6]. Relapsing CNS peritonitis suggests that the PD catheter has biofilm formation, and removal of the catheter should be considered [7]. The characteristics of CNS infections are increasing rates of antibiotic resistance. Colak documented that approximately 70% of methicillin-resistant *coagulase-negative staphylococci* (MRCNS) are involved in CNS peritonitis [8]. A previous study also identified methicillin resistance as an independent predictor of failure in CNS caused PD-related peritonitis [8].

In S*taphylococci*, the presence of PBP2a, encoded by the *mecA* gene, is responsible for resistance to β-lactam antibiotics [9]. This gene, known as the staphylococcal cassette chromosome mec (SCC*mec*), is related to a mobile genetic element (MGE) [10]. The unique combination of the *mec* gene complex class and the cassette chromosome recombinase (*ccr*) gene complex type determines the type of the SCC*mec*, and its variation within the junctional- (J-) regions determines the subtypes of each SCC*mec* type [11]. Currently, two unique *ccr* gene complexes have been described based on the assemble of *ccr* genes. One carries two adjacently located *ccr* genes, *ccrA* and *ccrB*, and the other carries *ccrC*. The *ccrA* and *ccrB* genes in *S. aureus* strains have been classified into four and five subtypes respectively, which lead to six *ccr* gene complex types referred to as type 1 (*ccrA1B1*), type 2 (*ccrA2B2*), type 3 (*ccrA3B3*), type 4 (*ccrA4B4*), type 7 (*ccrA1B6*) and type 8 (*ccrA1B3*). In contrary, all *ccrC* variants were identified with a high nucleotide similarity and derived from two allotypes, *ccrC1-2*,which constitute types 5 and 9 of the *ccr* gene complex [12]. To date, 14 different types of SCC*mec* have been identified with a number of different types and variations [13]. These cassettes are characterized by a high degree of diversity in the sequence and in the mobile elements that are integrated into them.

Phenol-soluble modulins (*psm*)-*mec* is a peptide that is excreted in small amounts. They are carried on three SCC*mec* cassettes (types II, III and VIII) which contain the class A *mec* gene complex [14]. *Psm* is a small peptide toxin that is considered to be a key virulence determinant in promoting pro-inflammatory processes and human cell lysis [15]. Moreover, some *PSMs* promote biofilm formation. Previous studies have also documented the existence of *psm-mec* gene in clinical isolates of methicillin-resistant *S. epidermidis* (MRSE) [16]. In addition, most MRSE have strong biofilm formation ability. Therefore, we speculate that *psm-mec* may also control biofilm formation of CNS isolates from patients with CNS PD-associated peritonitis. However, no relevant reports have been available until now. The aim of this study, was to investigate the role of *psm-me*c in CNS isolates from patients with CNS PD-related peritonitis biofilm formation, we evaluated 10 genetically unrelated CNS isolates from patients undergoing CNS PD-related peritonitis in relation to SCC*mec* types I, II and III; associated genes; and biofilm formation and the existence of *psm-mec* to understand the type of SCC*mec* among *staphylococci* to discover from which species different elements.

## Methods

### Patients

Between July 2019 and September 2021, 10 PD patients were recruited at Haikou People’s Hospital. The inclusion criteria as previously described were as follows [17]: (1) patients over 18 years old; (2) Continuous treatment with PD for more than 3 months and a stable clinical condition during the period of observation; (3) patients with CNS PD-related peritonitis during their time on PD; and 4) informed approval. PD patients with recent use of antimicrobials, previous hospitalization, tuberculosis, cirrhosis, severe hepatitis and tumor, were excluded.

### Study defections

The diagnosis of CNS-associated peritonitis was made as previously described on the basis of three of the following criteria [7]: (1) the presence of abdominal pain or the cloudiness of PD effluent; (2) white blood cell count in dialysis fluid > 100/µL with > 50% polymorphonuclear cells;(3) PD effluent (PDE) CNS positive culture. At least one of the first two criteria was present, and the third item was only required condition.

### Laboratory tests

For all patients, demographic data and clinical indicators were collected. These indicators were surveyed on admission.

### Microbiology tests

The microbiological tests were performed by centrifuging 50 mL of PDE was centrifuged at 3000 × g for 15 min and the pellet was seeded into Bact/Alet aerobic and anaerobic flasks (BioMerieux, Durham, NC, USA).The bacteria isolation and culture were performed in according with “National Clinical Practice Guidelines”. CNS were detected by using the VITEK − 2 automatic bacterial identification system and MALDI Biotyper Microflex LT/SH instrument (Bruker Daltonik GmbH, Bremen, Germany). Thus, CNS were instantly frozen at − 80 °C for testing.

### Antimicrobial susceptibility test AND screening MRCNS

Drug sensitivity testing was performed using the VITEK − 2 MIC fully automatic bacterial identification instrument. The latest American CLSI (Clinical & Laboratory Standards Institute) test method and criteria were used. All isolates with a minimum inhibitory concentration (MIC) to oxacillin at a dose of ≥ 0.5 µg/mL were taken as MRCNS and ≤ 0.25 µg/mL as methicillin-sensitive CNS (MSCNS). The quality control strain used was *Staphylococcus aureus*.

### Identification of CNS with biofilm formation

There were 2 cases of relapsing CNS peritonitis in this study. According to the 2016 ISPD recommendations, these cases should be considered CNS with biofilm formation [7]. Two strains of relapsing CNS peritonitis have been verified as the same source by MLST.

### DNA extraction from

The template was prepared by selecting 5 colonies to be assayed, which were resuspended in 500 µL of buffer heavy suspension cells and 50 µL of lysozyme. This suspension was heated in a water bath at 37 °C for 60 min and then centrifuged at 10,000 rpm for 1 min.

### Determination of the *mecA* gene and SCC *mec* typing

The *mecA* gene was detected in all isolates using the polymerase chain reaction (PCR) method with the PCR primers showed in Table 1. The amplification was carried out in a volume of 25 µL of a total reaction mixture comprising 30 ng of template DNA, 12.5 µL of PCR supernatant and 1 µL of the following primer. The PCR conditions for amplifying prokaryotic 16 S fragments were an initial denaturation at 95 °C for 3 min; 35 cycles of a denaturation at 95 °C for 30 s, an annealing at 55 °C for 30 s and an extension at 72 °C for 1 min; and a completion extension at 72 °C for 5 min. 2% agarose gel electrophoresis was used to verify PCR products. To eliminate the possibility of false-positive PCR results, ultrapure water was substituted for sample solution as a negative control throughout the DNA extraction process. SCC*mec* types were determined for all CNS isolates by evaluating the *mec* and *ccr* gene complex. The typability of the SCC*mec* cassettes has been defined in the following way: (1) Typeable (T) SCCmec cassettes were those where *ccr*, *mec* complex type and/or SCC*mec* were determined; (2) Non-Ascribed (NA) SCC*mec* types were defined as those with a novel combination of *ccr*, *mec* complex and/or SCC*mec*. and (3) Non-typeable (NT) were those not positive with the primer according to the schedule. Those within the NA and NT categories were defined as New SCC*mec* [18].

**Table 1:** Primers used for the classification of SCC*mec* type

Target	Primer direction	Nucleotide sequence 5’ to 3’	Amplicon size
*mecA*	Forward Reverse	5’-TCGTGTCACAATCGTTGACG-3’ 5’-TTATTTAACCCAATCATTGCTG-3’	356 bp
*SCCmec 1*	Forward Reverse	5’-CGAGTTGCTGATGAAGAAGG-3’ 5’-TTACCACAAGGACTACCAGC-3’	490 bp
*SCCmec 3*	Forward Reverse	5’-CCTACCTTAGTTGTCGTAAC-3’ 5’-CCATATTGTGTACGATGCGC-3’	283 bp
*mec-Class A*	Forward Reverse	5’-TCGTTATAAGTGTACGAATGG-3’ 5’-AATCGTGAATCACAAAGAAGC-3’	312 bp
*mec-Classs B*	Forward Reverse	5’-GGCGTCTAACTTGAACTCTC-3’ 5’-CAATGATGGACAATGACTGTG-3’	414 bp
*mec-Class C2*	Forward Reverse	5’-GGTGTAGAAGGTGTTATCATC-3’ 5’-GTCTCATCAATACGCCATTTG-3’	390 bp
*ccr*-1	Forward Reverse	5’-GAGTAACACCACAAACACAC-3’ 5’-GTCTGGATTGTCTTCGATGG-3’	320 bp
*ccr*-2	Forward Reverse	5’-ATCGGGTAAGCTCATGTTAC-3’ 5’-TGCTTACCTTCAGCTATCAC-3’	428 bp
*ccr*-3	Forward Reverse	5’-GGTACCAAGAAGCGAATACG-3’ 5’-GTCCGGATTATCTTCAATGG-3’	293 bp
*ccr*-4	Forward Reverse	5’-AGTGAAGTGCTCGACACTTG-3’ 5’-AACCTTCAATAGCACGTTGT-3’	878 bp
*ccr*-5	Forward Reverse	5’-AGTCTATAAAGGGTATGTCAG-3’ 5’-ATTACTATGACAGGCAGAACT-3’	354 bp
*psm-mec*	Forward Reverse	5’-GCATATGGATTTCACTGGTG-3’ 5’-ATGCAAAAGGACTGGAGTCC-3’	423 bp

### Sequence alignment

The *mecA* genes were recovered from the overlapping regions after partial sequencing using BioEdit software. The mecA sequence from NZ_CP013911.1 (S167) was used as a reference and aligned with CLUSTALW as implemented in MEGA 6 (www.megasoftware.net). To check for mutations, the aligned sequences and their encoded proteins were then further analyzed.

### Detection of *psm-mec*

The PCR was conducted on a Thermocycler (Sensoquest GmbH, Göttingen, Germany). The PCR primers were showed in Table 1. *Psm-mec*-for and *Psm-mec*-rev were used to detect the *psm-mec* gene.

### Statistical analysis

SPSS 23.0 software package was used for data analysis. The data were expressed as the mean ± SD and comparisons were made by t-test. The χ^2^ or Fischer’s exact test was performed for all comparisons. Differences of *P* < 0.05 were considered statistically significant.

## Results

### Participant characteristics

Between July 2019 and September 2021, 10 CNS PD-related peritonitis patients were enrolled There were 6 males and 4 females in the group. The average age was 56.17 ± 7.88 years, and the PD vintage was 27.5 ± 14.91 months. The pathogens were *S. caprae* in 3 patients, *S. epidermidis* in 4 patients, *S. haemolyticus* in 2 patients and *S.capitis* in 1 patient. The group was divided into two groups on the basis of the results of the MIC to oxacillin: the MRCNS and MSCNS groups. The biofilm formation group (BFG) and control group were formed. The BFG included CNS PD-related peritonitis patients with biofilm formation, while the control group was non-biofilm formation group (NBFG). A total of 6 MRCNS isolates and 2 BFG isolates were detected and confirmed to species in this study. In addition, we also found that there were more isolates within the NBFG were more in the MRCNS group than in the MSCNS group, although there was no significant difference (p = 0.053) (Table 2). The clinical characteristics and demographic of the patients are presented in Table 3.

**Table 2:** The correlation between the biofilm formation group and the MSCNS and MRCNS groups

	MSCNS(*n* = 4)		MRCNS(*n* = 6)		χ^2^ P
	Positive Isolate	Rate(%)	Positive Isolate	Rate (%)	
BFG	2	50	0	100	3.75 0.053
NBFG	2	50	6	0	

*BFG *Biofilm formation group, *NBFG *Non-biofilm formation group, *MSCNS *Methicillin-sensitive, *CNS *MRCNS Methicillin-resistant CNS

**Table 3:** Demographic and clinical characteristics of patients who underwent coagulase negative staphylococcus between MSCNS and MRCNS, and BFG and NBFG

Characteristics	MSCNS	MRCNS	P	BFG	NBFG	P
Age (years)	55.5±7.33	49.33±19.98	0.577	50.67±2.89	54.3±16.59	0.721
Gender(male/female)	2/2	5/1	0.17	1/1	6/2	0.033
Etiology of ESRD [n]			0.725			0.018
Glomerulonephritis	2	3		2	4	
Diabetic nephrology	2	3		0	4	
PD vintage (months)	28.25±14.06	47.67±37.23	0.355	40±3.46	43.1±37.73	0.893
Type of *Staphylococcus*	10		0.005	10		0.008
***Staphylococcus caprae***	3			1	1	
*Staphylococcus epidermidis*	1	3		1	4	
*Staphylococcus haemolyticus*	0	2		0	2	
*Staphylococcus capitis*	0	1		0	1	

*MSCNS *Methicillin-sensitive CNS, *MRCNS *Methicillin-resistant CNS, *BFG *Biofilm formation group, *NBFG *Non-biofilm formation group

### Antimicrobial susceptibility of CNS

All CNS isolates were checked for susceptibility against 9 antibiotics using the disc diffusion test.6 out of 10 (60%) and 9 out of 10 (90%) isolates were resistant to oxacillin and penicillin respectively. Six isolates were resistant to erythromycin, whereas 4 (40%) isolates showed resistance towards erythromycin in the MRCNS group. Three (30%) isolates were resistant to azithromycin, and three (30%) were resistant to clarithromycin (Table 4). MRCNS isolates were resistant to azithromycin, followed by 2 (2/10; 20%) to clarithromycin, 1 (1/10; 10%) to cotrimoxazole, and 2 (2/10; 20%) to rifampicin and moxifloxacin (Table 4). Interestingly, in the BPCNS group the resistance rates towards oxacillin (0%) were comparatively low compared with BNCNS isolates, which exhibited a resistance towards oxacillin of 75% (p = 0.053) (Table 4).

**Table 4:** Antibiotic resistance pattern of MSCNS and MRCNS, and BFG and NBFG

	MSCNS(*n* = 4)		MRCNS(*n* = 6)		χ^2^	P	BFG(*n* = 2)		NBFG(*n* = 8)		χ^2^	P
	Positive Isolates	Rate(%)	Positive Isolates	Rate (%)			Positive Isolates	Rate(%)	Positive Isolates	Rate (%)		
Oxacillin	0	0	6	100	10	0.002	0	0	6	75	3.75	0.053
Penicillin	3	75	6	100	1.667	0.197	2	100	7	87.5	0.278	0.598
Erythromycin	2	50	4	66.7	0.278	0.598	1	50	5	62.5	0.104	0.747
Azithromycin	1	25	2	33.3	0.079	0.778	1	50	2	25	0.476	0.49
Clarithromycin	1	25	2	33.3	0.079	0.778	1	25	2	25	0.476	0.49
Cotrimoxazole	1	25	1	16.7	0.104	0.747	0	0	2	25	0.625	0.429
Rifampicin	0	0	2	33.3	1.667	0.197	0	0	2	25	0.625	0.429
Moxifloxacin	0	0	2	33.3	1.667	0.197	0	0	2	25	0.625	0.429
Levofloxacin	1	25	2	33.3	0.079	0.778	0	0	3	37.5	1.071	0.301

*MSCNS *Methicillin-sensitive CNS, *MRCNS *Methicillin-resistant CNS, *BFG *Biofilm formation group, *NBFG *Non-biofilm formation group

### Types of SCC *mec* types and multiple elements of SCC *mec*

**Fig. 1:**
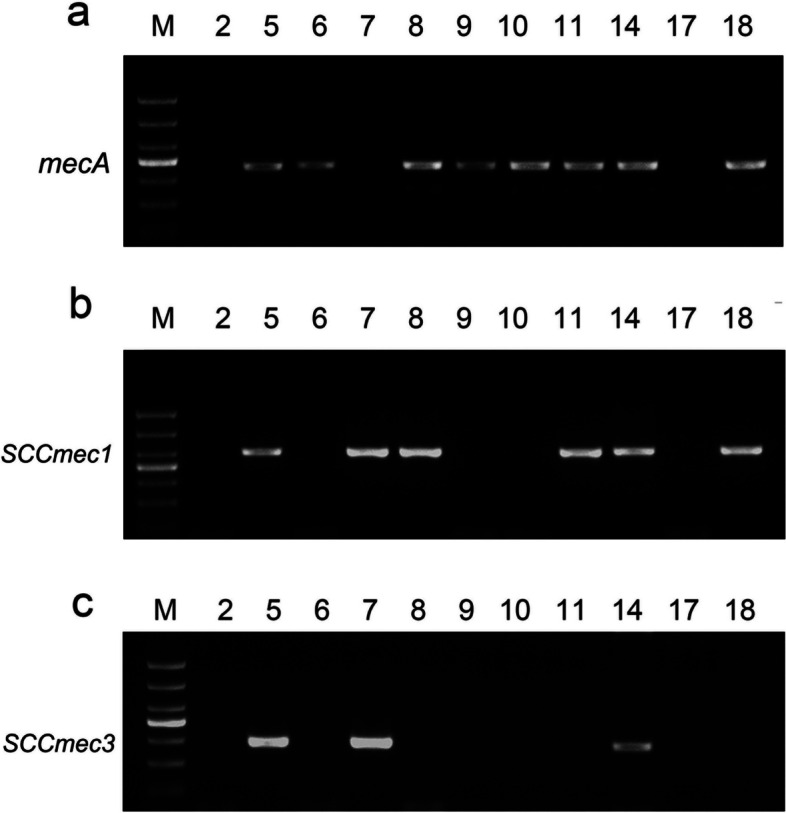
**a** The expression of *mecA* in CNS isolates. **b** The expression of SCC*mec*1 in CNS isolates. **c** The expression of SCC*mec*3 in CNS isolates. M: Marker. 5, 8, 10–14, 18: MRCNS isolates. 2, 7, 9, 17: MSCNS isolates. 6, the reference strain of *Staphylococcus aureus*

The *mecA* gene was tested in 7/10 (70%) CNS isolates, 6/6 (100%) MRCNS isolates (Fig. 1a) and 7/8 (87.5%) NBFG isolates (Table 5). SCC*mec* types were fully determined in 10 isolates. Table 5 (Fig. 1a, b) shows the SCCmec typing results of these isolates. Those with two *mecA* classes were referred to as CNS with multiple SCC*mec* elements; CNSs with a single SCC*mec* element were considered to be those with one class of *mecA* complex and one class of *ccr* complex. Moreover, a new single SCCmec type was recovered only from 1 isolate (Table 6). Seven *staphylococci* (70%) carried SCC*mec*, followed by 4 isolates carrying a single SCC*mec* type I (40%) (Fig. 1b). Surprisingly, multiple SCC*mec* elements in 30% (3/10) of isolates. In particular, all isolates harboring five *ccr* complexes. Isolates had multiple SCCmec elements, especially all five *ccr* complexes. In all, 3 SCC*mec* cassettes were either NT or NA (30%) (Table 5). Of the 6 MRCNS isolates, 3 carried a single SCC*mec* type I (50%) and 2 isolates carried multiple SCC*mec* types (33.3%) (Table 5; Fig. 1c).

**Table 5:** SCC *mec* typing from 10 CNS isolates recovered from patients with *coagulase-negative staphylococci* peritoneal dialysis-related peritonitis

Isolate number	Identification	*mecA* PCR	SCC*mec*	*ccr* type	*mecA* class	Character Of SCC*mec*	BFG isolates	MRCNS
1	*S. epidermidis*	+	I+III	Type 3+5	Class A	T	N	Y
2	*S. epidermidis*	+	I	Type 1+4	Class B	T	N	Y
3	*S.caprae*	-	/	Type 4	/	NA	Y	N
4	*S. haemolyticus*	+	/	/	/	NT	N	Y
5	*S. haemolyticus*	+	I	Type 2+5	Class C2	T	N	Y
6	*S. epidermidis*	+	I+III	Type 1+2+4	Class B	T	N	Y
7	*S. capitis*	+	I	Type 3(A3B3)	Class A	T	N	Y
8	*S.caprae*	+	I	Type 4	/	T	N	N
9	*S. epidermidis*	-	I+III	Type 2+5	/	T	N	N
10	*S.caprae*	-	/	/	/	NT	Y	N

*S* *Staphylococci, T *Typeable SCC*mec, NA *Non-Ascribed (NA) SCC*mec* types were those with a novel combination of *ccr*, *mec* complex, and/or SCC*mec*; NT: Non-Typeable (NT) SCC*mec* types were considered those that did not yield positive results with the primer sets used, per scheme; Y: Yes; N: No

**Table 6:** Detection of *mecA* segments and SCC*mec* typing results of CNS isolates

Character Of SCC*mec*	Identification	*mecA* PCR	SCC*mec*	*ccr* type	*mecA* class	MRCNS
New indentified single SCCmec tpye	*S.caprae*	-	New	Type 4	/	N
Isolates without mecA detected	*S.caprae*	-	/	Type 4	/	N
	*S.caprae*	-	I	Type 4	/	N
	*S. epidermidis*	-	I+III	Type 2+5	/	N
	*S.caprae*	-	/	/	/	N

*New *New SCC*mec* were defined as those enclosed within NA and NT categories; Y: Yes; N: No

Unexpectedly, among these 4 CNS strains with no *mecA* segment detected, 2 strains contained a *ccr* complex (Table 6). In addition, 1 strain showed multiple *ccr* complexes. In this study, a grand total of 14 *ccr* complex segments were successfully aligned. No new ccr allotypes or new ccr alleles have been determined.5 isolates (50%) showed more than one type of *ccr*. The 2 *ccrA1B1*, 3 *ccrA2B2*, 2 *ccrA3B3*, 4 *ccrA4B4* and 3 *ccrC1* alleles from all CNS isolates were matched based on a BLAST search of GenBank sequences. Additional *ccr* genes (*ccrA3B3*, *n* = 1; *ccrA2B2*, *n* = 2) were also present in three SCC*mec* cassettes carrying *ccrC1* (Table 5).

### *MecA* sequencing and alignment

High homology with the *mecA* gene of the *S. haemolyticus* reference strain S167 was found in CLUSTALW (MEGA 6) among the 7 *mecA* genes sequenced from CNS isolates, However, no mutations were found (Table 5 and Supplemental material 1).

### Detection of *psm-mec*

The *psm-mec* gene was detected in 2/10 (20%) CNS isolates. The pathogens were *S. epidermidis* and *S.capitis*. Moreover, they were all NBFG isolates. The phenotype of two isolates carrying *psm-mec* have been tested by ViteK2. We have observed their phenotypes were all MRCNS strains with multiple resistance. Furthermore, the phenotype is consisting with the genotype. Interestingly a combination of the class A *mec* complex and *ccr* type 3 was found in all these isolates. One of the two *psm-mec* positive isolates harboring SCC*mec* type I + III, accompanied by a combination of *ccr* types 3 and 5 and class A *mec* complex (Table 7). The *psm-mec* alleles were assigned on the basis of a BLAST search with sequences in GenBank, which revealed a homology of 99%, as shown in Supplemental material 2. There was no different the between two *psm-mec* alleles (Supplemental materials 3 and 4).

**Table 7:** SCCmec typing results for *coagulase negative staphylococci* peritonitis with *psm-mec* positivity

Staphylococcal isolate (s)	*mec* A	SCC*mec* type	ccr type	mec class	No. of Isolates
*S. epidermidis*	Positive	I + III	Type 3 + 5	Class A	1
*S.capiti*	Positive	I	Type 3	Class A	1

## Discussion

As far as we know, few studies have explored the correlation between the elemental compositions of SCC*mec* and the presence of *psm-mec* in CNS isolates from patients with CNS peritonitis. In the current study, the most common CNS in PD-related peritonitis was *S.epidermidis*, followed by *S.caprae*, *S.haemolyticus* and *S.capitis*. *S. epidermidis* are the most prevalent pathogens worldwide [19]. In agreement with previous studies, we identified *S. epidermidis* as the predominant *Staphylococcus* species in patients with CNS PD-related peritonitis, accounting for 50% of cases. There were cases of 2 relapsing CNS peritonitis in this study. The pathogen in both cases was all *S.capitis*. A total of 6 MRCNS isolates and 2 BFG isolates were recovered. We also describe the antimicrobial resistance patterns and genetic variability of CNS in these patients. The resistance of CNS towards penicillin is well established. Rafatpanah et al. [20] revealed that 70% of recovered *S. epidermidis* isolates were resistance to penicillin. In accordance with their findings, we found that 90% of CNS isolates were genotypically resistant to penicillin. In the case of methicillin-resistant *staphylococci*, the *mecA* gene is involved in conferring resistance to almost all β-lactam molecules. Our study demonstrated that the resistance of CNS isolates to β-lactam antibiotics was higher. It is suggested that CNS peritonitis is characterized by a high rate of resistance to β-lactam antibiotic in our center. The *mecA* gene is determined methicillin-resistance in *staphylococci*, which is present in the SCC*mec*, leading to a reduced affinity for β-lactam antibiotics [10].

In general, the involvement of various types of SCC*mec* in the CNS depends on the geographical locations and host species [21]. In our study, SCC*mec* typing allowed the differentiation between types I-III among the CNS strains. Our results showed a high rate of SCC*mec* I (40%), followed by SCC*mec* I + III (30%) (multiple SCC*mec*). Of the 6 MRCNS isolates, 3 isolates were carrier of SCC*mec* type I (50%), and 2 isolates were carrier of SCC*mec* type I + III (33.3%). SCC*mec* types I, II and III are most frequently associated with nosocomial infections resulting from methicillin-resistant *staphylococci* [22]. However, SCC*mec* types IV and V are predominantly associated with community-acquired (CA) infections, SCC*mec* types I, II and III are markedly larger than the other types. It is suggested that the majority of isolates were hospital-associated (HA) CNS isolates in our study, especially for MRCNS. PD is basically a home treatment. Our findings therefore represent an interesting epidemiological situation, as the main risk factors associated with the development of drug-resistant bacteria (previous hospital admission, history of antimicrobials use) were not evident in our PD patients [19]. We assumed that during frequent outpatient visit, routine consultations and some PD-related procedures of PD patients exposure to the hospital environment, may facilitate colonization by the hospital-associated CNS, leading to peritonitis and reaching the peritoneum within the dialysis procedure via intraluminal pathway or the periluminal (catheter-skin interface). Types I-III are relatively large in size and carry several antibiotic resistance determinants. Therefore, it can be interpreted that the CNS containing SCC elements in this study was also MRCNS. Types IV and V have been defined as new versions of SCC*mec*, which are short in length and generally do not carry any antibiotic resistance genes in association with the *mec*-gene complex [23]. Unfortunately, we did not detect types IV and V SCC*mec* in this study.

In the last few years, more novel SCC*mec* elements, including non-*mecA*-encoding cassettes have been discovered in CNS. Surprising among these 4 CNS strains, one was now detected a *mecA* segment, but they were all MSCNS strains. Only 1 isolate carrying a new single SCC*mec* type was then examined in our study. There were 2 cases of relapsing CNS peritonitis in this study. However, they were all MSCNS strains. The absence of the SCC*mec* element was found in 1 isolate and 1 isolate with only *ccr* 4. The absence of the SCC*mec* element in CNS isolates may indicate extreme polymorphism [24]. Samples containing only *ccr* or *mec* may be subject to recombination of the SCC*mec* element, insertion of a new structure, or structural re-arrangements [25]. Furthermore, it is suggested that these 2 isolates are community-acquired (CA)-CNS strains based on the MSCNS isolates with new SCC*mec*. Szczuka et al. reported in *S. aureus*, *S. epidermidis* and other CNSs multiple copies of the *ccr* complex have been observed. However, the majority are a combinations of *ccrAB* and *ccrC* [26]. Interestingly, the present study revealed different combinations of *ccr* types. 5 isolates (50%) showed more than one type of *ccr*. Furthermore, more than 1 *ccr* were also copresent in most of MRCNS, indicating that the clustering of the two *ccr* genes may confer an adaptive benefit. We observed three SCC*mec* cassettes with *ccrC 1* that carried additional *ccr* genes (*ccrA3B3*, *n* = 1; *ccrA2B2*, *n* = 2). Few studies have explored a heterogeneous combination of *ccr* complexes in CNS strains, in particular all of the five types of *ccr* complexes that exist in the CNS strains from PD patients. Therefore, it was not unexpected that these CNS strains contained multiple SCC*mec* elements. The incidences of multiple SCC*mec* elements were as high as those observed in our study. For unveiling the actual presence and functionality of redundant *ccr* genes, and under specific conditions whether this indicates an adaptation advantage further analyses should be performed.

*Psm-mec* is not restricted to *S. aureus*, it has also been used to purify it from the culture suspension of MRSE. In particular, the gene *psm-mec* has been detected in other CNS [27]. In present study the *psm-mec* gene was identified in 2/10 (20%) CNS isolates. The pathogens were *S. epidermidis* and *S.capitis*. Moreover, these isolates were all MRCNS. The results of this study revealed that the *psm-mec* gene was widespread within MRCNS strains. The existence of the *psm-mec* signal in the MRCNS spectra tested in our study indicates the existence of certain SCC*mec* cassettes which confer methicillin resistance. Consistent with previous studies, we found that 2 isolates with *psm-mec* positivity carried a combination of the class A *mec* complex and *ccr* types 3 and 5. More detailed studies showed that the *psm-mec* locus has been observed in SCC*mec* types II, IIA, IIB, IID, III, and VIII of a large number of *Staphylococcus* species [28]. However, the types of SCC*mec* with *psm-mec* in present study were type I and I + III. This is the first study in which *psm-mec* was found in SCC*mec* type I, suggesting that SCC*mec* type I is a possible marker for analytical purposes. In agreement with previous studies the several widely distributed hospital-associated methicillin resistant *S. aureus* (HA-MRSA) lineages have been found to carry the *psm-mec* locus [29]. CA-MRSA strains are genetically distinctive and significantly more invasive than HA-MRSA strains. Moreover, outside public healthcare institution these strains reveal elevated production of virulence factors, and are able to lead to disease from otherwise healthy individuals. Furthermore, CA-MRSA lacks the *psm-mec* locus. Our study suggested that these two isolates were HA-CNS strains based on their SCC*mec* types.

Interestingly, Qin et al. found CA-MRSA strains had a greater ability to disseminate on soft agar than HA-MRSA strains, and the deletion of the *psm-mec* locus in CA-MRSA strains was suggested to be responsible for this phenotype . Therefore, it will be possible to identify the origin of CNS harboring the *psm-mec* gene. PSM peptides have a significant effect on biofilm formation. *PSMs* lead to the structuring and biofilm maturation, which includes forming nutrient-transporting pathway [28]. In contrast, 2 *psm-mec* positive isolates were NBFG isolates in the current study. A study investigating the absence of *psm-mec* in a mouse skin infection model either had not significantly change in virulence ,or decreased virulence. In addition, *psm-mec* has only a very small effects expressing other *psm* peptides. Since the region around *psm-mec* is well preserved in class A SCC*mec* elements, the author consider that the type of SCC*mec* element cannot influence the role of *psm-mec* in virulence [30]. However, a recent study revealed that *psm-mec* may be involved as a virulence factor in *S. epidermidis*. This study was mainly on the basis of the study isogenous mutants of the *psm-mec* gene with clinical strains of *S. epidermidis* [29]. Unfortunately, no *psm-mec* mutation was found in our study.

This study had several shortcomings. First, the size of the sample was rather limited; then, larger sample sizes are required in further studies. Second, we were unable to identify which *ccr* complex was associated with the particular SCC*mec*, especially when carrying multiple SCC*mec*. Third, the existence of multiple and untypable SCC*mec* elements poses a major challenge to SCC*mec* typing in CNS. Whole genome sequencing of more CNS would help construct new typing method by understanding the exact composition of multiple SCC*mec* and the relative location and further clarify the function of *ccr* complexes in the dissemination of SCC*mec* elements throughout CNS.

In summary, this study was the first study to determine the of frequency and diversity of SCC*mec* types and the existence of *psm-mec* in CNS isolates from CNS PD-related peritonitis. The current study demonstrated a high prevalence of MRCNS harboring SCC*mec* type genes. The majority of isolates were hospital-associated isolates. Furthermore, the 2 isolates with *psm-mec* positivity were MRCNS in the NBFG. The SCC*mec* typing system is the major marker for distinguishing between CA-MRSA and HA-MRSA. The frequent exposure of such patients to hospitals is the main risk factor. Hence, strategies must be applied to prevent the spread of such pathogens in both community and hospital environments. As a result, additional large scale and comprehensive studies are needed to define the SCC*mec* type distribution and to determine the molecular epidemiological characteristics of *psm-mec* and its possible role in hospital and community infection in particular species. Additionally, more CNS samples are needed.

### Supplementary Information

**Additional file 1.**

**Additional file 2.**

**Additional file 3.**

**Additional file 4.**

## Data Availability

The datasets generated and analyzed during the current study are available from the corresponding author on reasonable request.
