# Supplementary material for: Identification and characterization of SCCmec typing with psm-mec positivity in staphylococci from patients with coagulase-negative staphylococci peritoneal dialysis-related peritonitis
Source: BMC Microbiol. 2023 Sep 23;23:267. doi: 10.1186/s12866-023-03017-2 (PMC10517493; doi:10.1186/s12866-023-03017-2)
Supplement: Supplementary file 1 — Additional file 1. [file 12866_2023_3017_MOESM1_ESM.docx]

**Sequence of mecA**

ATGTCTCGTCGCAATACATTAATAGAGAAAGAAAAAGATGGCAAAGATATTCAACTAACTATTGATGCTAAAGTTCAAAAGAGTATTTATAACAACATGAAAAATGATTATGGCTCAGGTACTGCTATCCACCCTCAAACAGGTGAATTATTAGCACTTGTAAGCACACCTTCATATGACGTCTATCCATTTATGTATGGCATGAGTAACGAAGAATATAATAAATTAACCGAAGATAAAAAAGAACCTCTGCTCAACAAGTTCCAGATTACAACTTCACCAGGTTCAACTCAAAAAATATTAACAGCAATGATTGGGTTAAATAAAGA
