# Supplementary material for: Identification and characterization of SCCmec typing with psm-mec positivity in staphylococci from patients with coagulase-negative staphylococci peritoneal dialysis-related peritonitis
Source: BMC Microbiol. 2023 Sep 23;23:267. doi: 10.1186/s12866-023-03017-2 (PMC10517493; doi:10.1186/s12866-023-03017-2)
Supplement: Supplementary file 4 — Additional file 4. [file 12866_2023_3017_MOESM4_ESM.zip › Legends of supplementary figure.docx]

Supplementary figure 1

Original figure of Figure 1a. The expression of *mecA* in CNS isolates

Supplementary figure 2

Original figure of Figure 1b. The expression of SCC*mec*1 in CNS isolates.

Supplementary figure 3

Original figure of Figure 1c. The expression of SCC*mec*3 in CNS isolates.
