# Supplementary figures and images for: Identification and characterization of SCCmec typing with psm-mec positivity in staphylococci from patients with coagulase-negative staphylococci peritoneal dialysis-related peritonitis
Source: BMC Microbiol. 2023 Sep 23;23:267. doi: 10.1186/s12866-023-03017-2 (PMC10517493; doi:10.1186/s12866-023-03017-2)

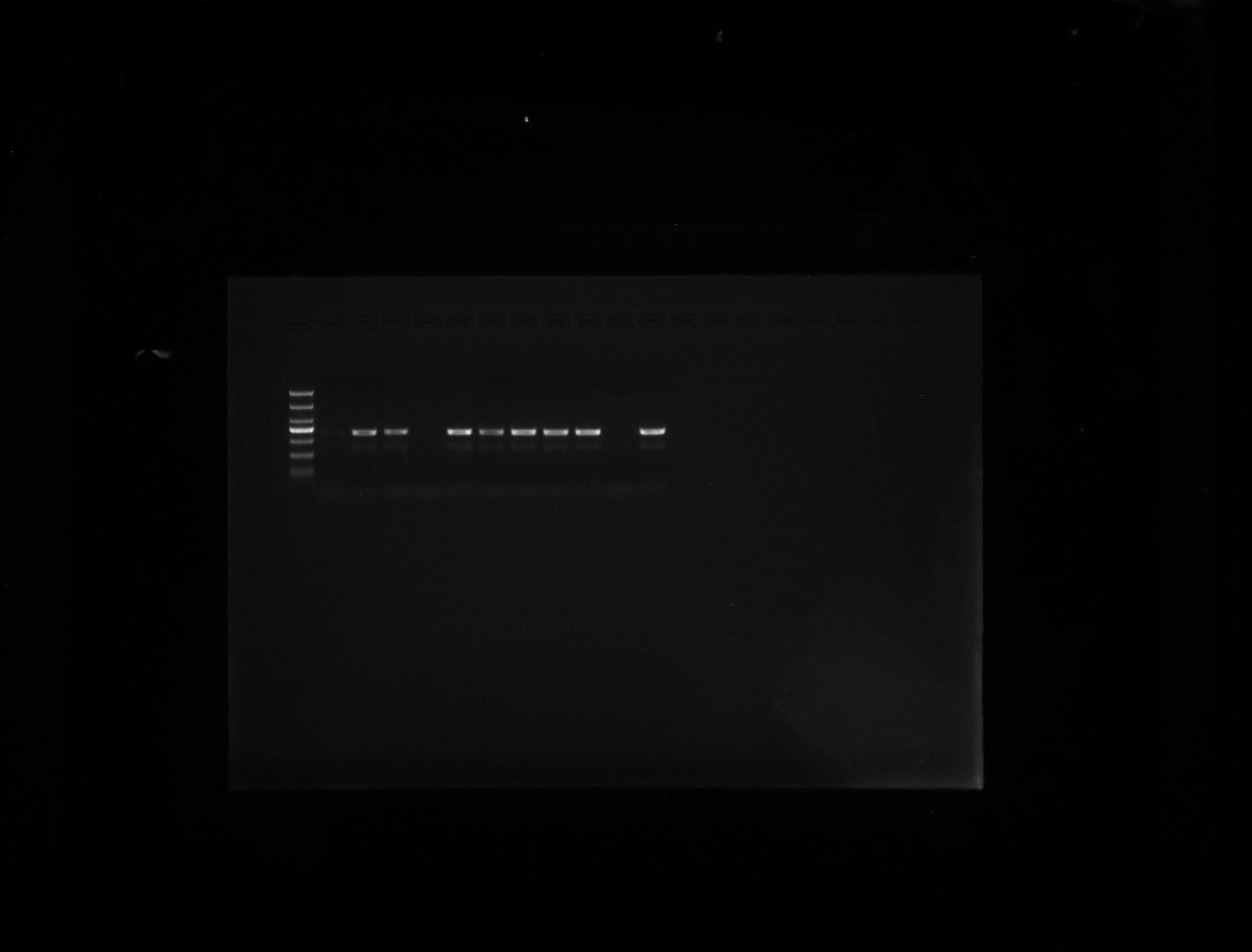

Supplement: Supplementary file 4 — Additional file 4. [file 12866_2023_3017_MOESM4_ESM.zip › Supplementary figure 1.jpg]

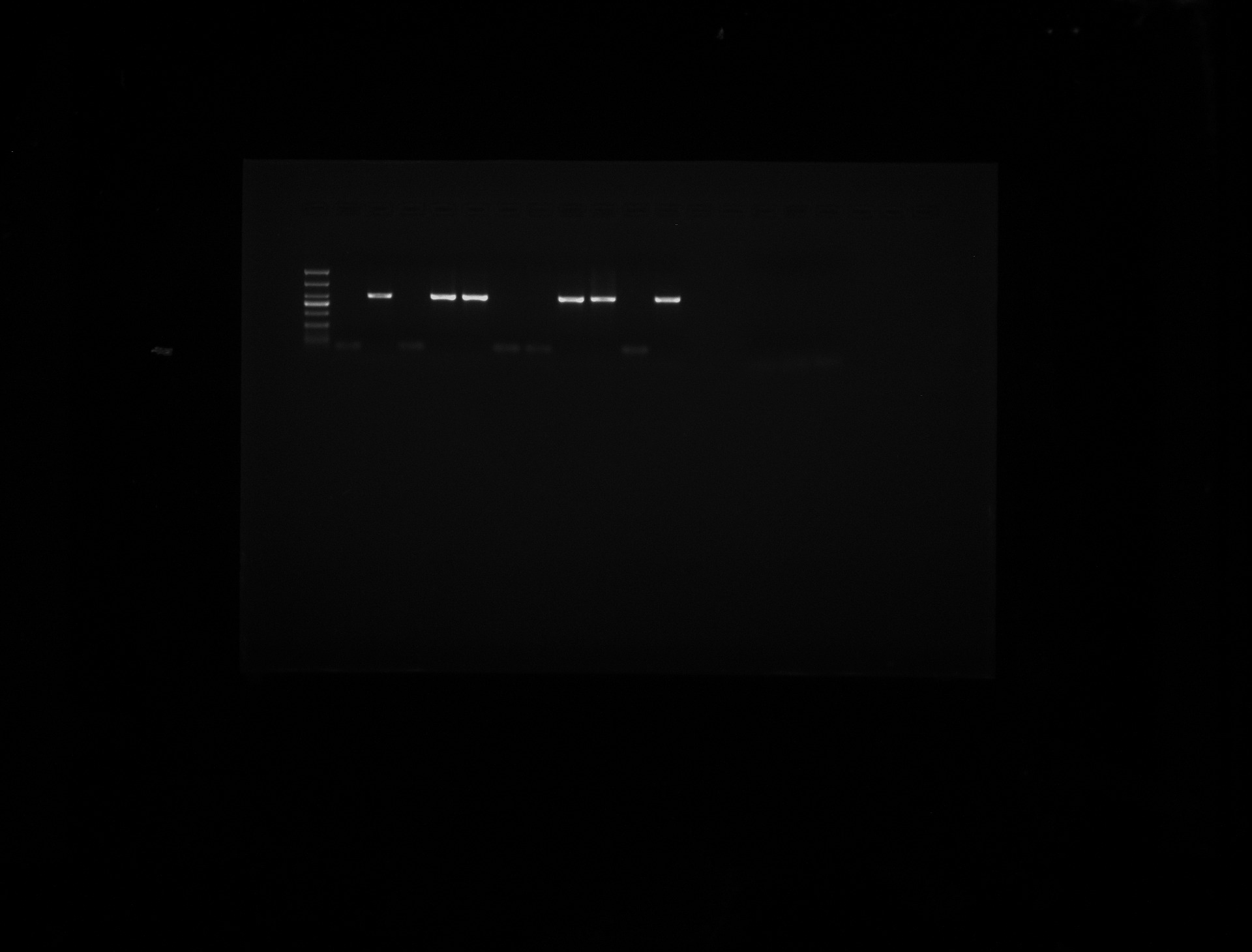

Supplement: Supplementary file 4 — Additional file 4. [file 12866_2023_3017_MOESM4_ESM.zip › Supplementary figure 2.jpg]

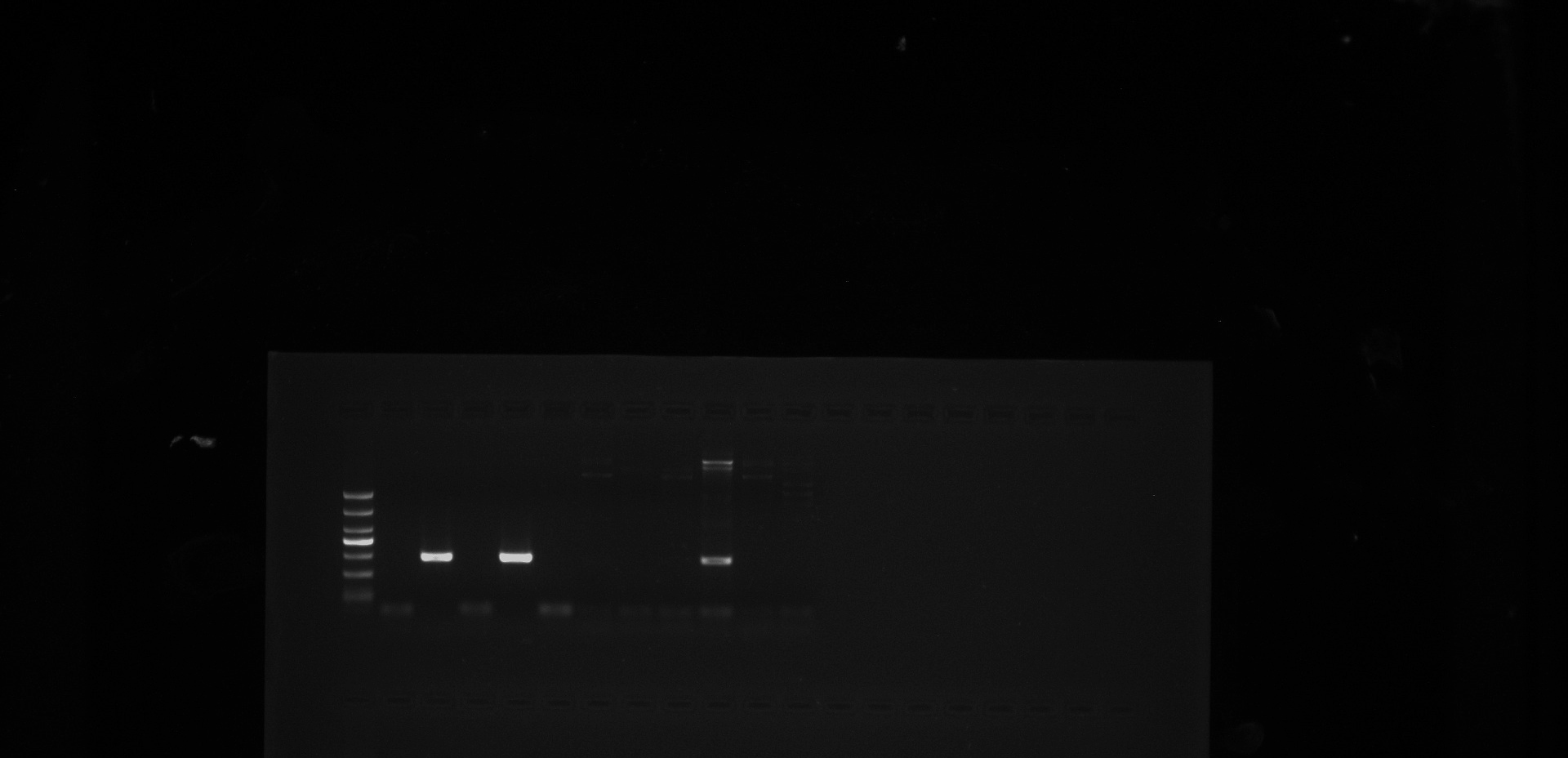

Supplement: Supplementary file 4 — Additional file 4. [file 12866_2023_3017_MOESM4_ESM.zip › Supplementary figure 3.jpg]
